# Teleneurology clinics for polyneuropathy: a pilot study

**DOI:** 10.1007/s00415-019-09553-0

**Published:** 2019-11-03

**Authors:** Andrew M. Wilson, Nasheed I. Jamal, Eric M. Cheng, Moira Inkelas, Debra Saliba, Andrea Hanssen, Jorge A. Torres, Michael K. Ong

**Affiliations:** 1 Department of Neurology, VA Greater Los Angeles Healthcare System, 10940 Wilshire Blvd., Suite 710, Los Angeles, CA 90024 USA; 2 Department of Neurology, David Geffen School of Medicine At UCLA, Los Angeles, CA USA; 3 Department of Health Policy and Management, Fielding School of Public Health At UCLA, Los Angeles, CA USA; 4 Geriatric Research, Education, and Clinical Center, VA Greater Los Angeles Healthcare System, Los Angeles, CA USA; 5 UCLA/Jewish Home Borun Center for Gerontological Research, Los Angeles, CA USA; 6 Department of Neurology, Massachusetts General Hospital, Harvard Medical School, Boston, MA USA; 7 Department of Neurology, Brigham and Women’s Hospital, Boston, MA USA; 8 Department of Medicine, VA Greater Los Angeles Healthcare System, Los Angeles, CA USA; 9 Department of Medicine, David Geffen School of Medicine At UCLA, Los Angeles, CA USA

**Keywords:** Telemedicine, Teleneurology, Neuropathy, Polyneuropathy, Evidence-based, Guidelines

## Abstract

**Introduction:**

Polyneuropathy (PN) is a common condition with significant morbidity. We developed tele-polyneuropathy (tele-PN) clinics to improve access to neurology and increase guideline-concordant PN care. This article describes the mixed-methods evaluation of pilot tele-PN clinics at three community sites within the Greater Los Angeles VA Healthcare System.

**Methods:**

For the first 25 patients (48 scheduled visits), we recorded the duration of the tele-PN visit and exam; the performance on three guideline-concordant care indicators (PN screening labs, opiate reduction, physical therapy for falls); and patient-satisfaction scores. We elicited comments about the tele-PN clinic from patients and the clinical team. We combined descriptive statistics with qualitative themes to determine the feasibility and acceptability of the tele-PN clinics.

**Results:**

The average tele-PN encounter and exam times were 28.5 and 9.1 min, respectively. PN screening lab completion increased from 80 to 100%. Opiate freedom improved from 68 to 88%. Physical therapy for patients with recent falls increased from 58 to 100%. The tele-PN clinic was preferred for follow-up over in-person clinics in 86% of cases. Convenience was paramount to the clinic’s success, saving an average of 231 min per patient in round-trip travel. The medical team’s caring and collaborative spirit received high praise. While the clinic’s efficiency was equal or superior to in-person care, the limited treatment options for PN and the small clinical exam space are areas for improvement.

**Conclusion:**

In this pilot, we were able to efficiently see and examine patients remotely, promote guideline-concordant PN care, and provide a high-satisfaction encounter.

## Introduction

Polyneuropathy (PN) is a common condition that affects 2–7% of the population [1]. People with PN suffer from higher functional impairments (e.g., trouble bathing, housekeeping) and mortality rates, even after controlling for associated comorbidities [2]. Prompt diagnosis and management of PN may stop the progression of disease or prevent complications through pharmacologic and safety interventions.

Despite practice parameters and quality measures to encourage evidence-based care [3, 4], the diagnosis and management of PN vary widely in the community. For example, Callaghan et al. noted over 400 patterns of testing for PN from 15 relevant tests [5]. Of concern was the frequency of ordering high-cost magnetic resonance imaging, which has a limited role in the evaluation of PN, and the infrequency of ordering low-cost, high-yield laboratory tests [i.e., a diabetes test, vitamin B12 levels, and serum protein electrophoresis with immunofixation (SPEP with IFE)] [5]. Providing access to neurologists appears to improve the delivery of evidence-based PN care [6].

Unfortunately, due to geographic or transportation barriers, as well as the paucity of neurologists in rural areas, an in-person neurological visit is not always feasible for some people with PN [7, 8]. One potential solution is to support primary care providers in the practice of evidence-based PN care with educational outreach, institutional protocols, and/or audit and feedback systems; however, these strategies have demonstrated only a small improvement in practice [9]. Therefore, we designed a teleneurology clinic for PN (i.e., a real time, remote audio–visual communication between a patient and a neurologist for PN care) to increase access to neurologists compared to in-person only models and thus enhance utilization of evidence-based care through the experts’ knowledge of current guidelines, translation of guidelines into realistic clinical policies, and tailoring of clinical policies to individual patients [10, 11].

This article describes the development and the mixed quantitative and qualitative evaluation of a pilot teleneurology clinic for PN (tele-PN clinic), evaluating its feasibility and acceptability within a large healthcare system. Our goals were threefold: (1) to determine the ability of the tele-PN team to efficiently see and examine patients (2) to increase PN guideline-concordant care, and (3) to provide a high-satisfaction encounter.

## Methods

### Institutional review

This study was approved as a program evaluation activity by the Greater Los Angeles Veterans Affairs (GLA VA) institutional review board.

### Setting

This pilot was conducted in the GLA VA healthcare system, the largest integrated healthcare organization within the VA. Neurologists are located at 3 of the 11 clinical sites. The novel GLA VA tele-PN clinics feature a neuromuscular specialist (NIJ) at the West Los Angeles VA hub site and a licensed vocational nurse telepresenter at the community-based outpatient center (CBOC) spoke site where the patient is visiting. The VA Clinical Video Teleconference platform uses a wired network with a bandwidth of 384 kilobits per second. Telemedicine equipment includes Cisco CTS-EX90-K9 (resolution: 640 × 360) at the hub site and GlobalMed media carts (resolution: 1920 × 1080) at the spoke sites.

Three CBOCs were selected as pilot spoke sites in the summer of 2017 based on a needs assessment for PN care and their telemedicine capabilities. Primary care providers and managers at all three sites were partners in this outreach effort, and we co-developed a service agreement for each site so that patients meeting criteria for the tele-PN clinic could be directly enrolled with patient consent without a referral from the patient’s primary care provider.

#### Patient selection for the tele-PN clinics

Patients who receive primary care from a spoke site and have a PN diagnosis in their active problem list (based on ICD10 codes of E08.4–E13.4, G60–65, G90 in the electronic medical record) are screened for the clinic by the clinic nurse or study author (AH or AMW, respectively). After chart review, patients are excluded from invitation to the tele-PN clinic if they meet any of the following three criteria: (1) they do not meet Toronto Consensus Panel criteria for polyneuropathy (i.e., there were no symptoms, no signs, and no electrodiagnostic tests supportive of PN) [12]; (2) they are asymptomatic in terms of pain and mobility on current treatment; or (3) they have another complicating neurologic condition requiring an in-person neurologic visit (e.g., Parkinson’s disease). Table 1 summarizes the eligibility screening of patient selection for the tele-PN clinics. As of November 1, 2017, we had screened a total of 188 patients, 116 (62%) of which were deemed eligible for the tele-PN clinics.

**Table 1:** Eligibility screen for the tele-PN clinics

	Count
Total patients with PN diagnosis	275
Patients reviewed/screened	188
Not PN	27 (14%)
Asymptomatic	16 (9%)
Complicated	29 (15%)
Eligible for tele-PN clinic	116 (62%)

As of November 1, 2017, there were 100, 119, and 56 patients with an active ICD10 diagnosis of polyneuropathy at the three participating VA clinics for a total of 275 patients. Of those, 188 patients have had their medical record screened by the nurse coordinator or study investigator. Rows 4–6 list the reason and counts (and percentages) of the reviewed patients for exclusion from the tele-PN clinic. The final row lists the number (and percentage) of the reviewed patients who were deemed eligible for the tele-PN clinic

Eligible patients are contacted by the study author to see if they would be interested in a teleneurology visit for PN. This visit is either a new neurology visit or a substitute for the in-person visit if the Veteran is already receiving neurologic care. For the pilot, patients who were on multiple neuropathic medications, were having falls, or had been lost to neurology follow-up were prioritized to be scheduled first per stakeholder request. Stakeholders included the primary care providers, neurologists, and the tele-PN team. We included the first 25 patients as part of the pilot evaluation. Patients were scheduled in typical fashion with appointment reminders by mailed card and a phone call 3 days prior to the visit. Encounters were scheduled for 1 h for new patients and 30 min for return visits.

The three tele-PN clinics were established between August 2017 and February 2018, and we enrolled 11, 9, and 5 unique individuals, respectively, at each site, during the pilot phase. Each clinic site runs for 2 consecutive hours per month with one teleneurologist and one telepresenter.

### Intervention

#### Tele-PN clinic encounter description

**Fig. 1:**
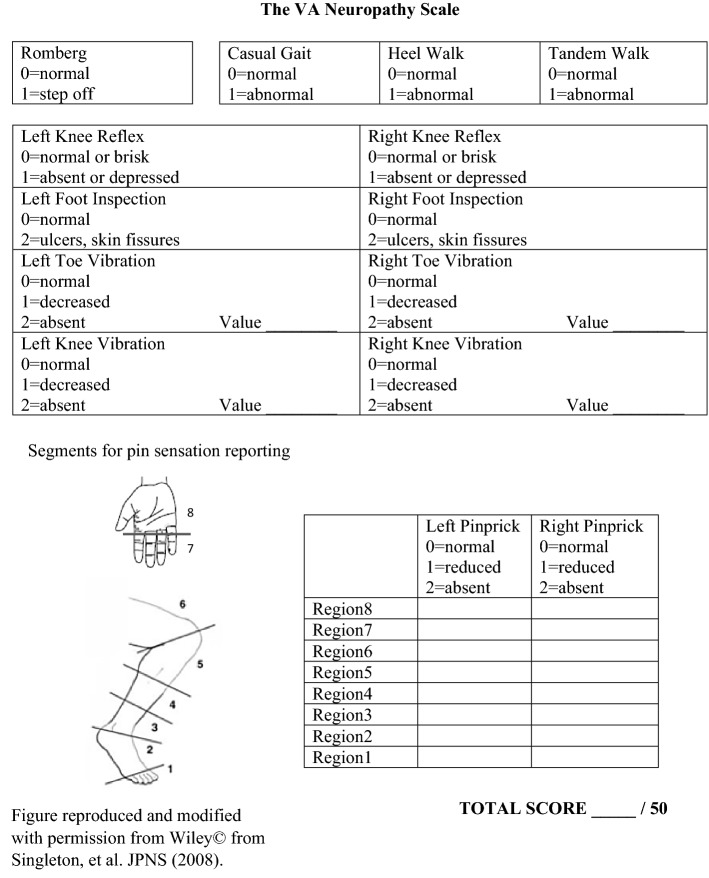
The VA Neuropathy Scale

During the encounter, patients undergo a check-in process, vitals, a specialized neuromuscular exam (the VA Neuropathy Scale [VANS]), and the history prior to discussing the diagnosis and treatment plan. These discussions are similar to those of in-person visits. The VANS (Fig. 1) was developed by the authors (AMW, NIJ) specifically for the tele-PN clinic by modifying existing, validated neuropathy screening instruments [13–21]. It has abbreviated balance, gait, reflex, and sensory testing that can be observed remotely by the neurologist [22].

The study author trained two telepresenters at each site to perform the VANS. Training was performed on-site at each CBOC for approximately 1 h and consisted of the proper use of exam tools and exam techniques, a demonstration of the full VANS by the neurologist on the telepresenters acting as a patient, and several “mock” VANS exams by the telepresenters on the neurologist who simulated common exam situations. VANS exam technique was reinforced during clinical encounters. During the on-site training visit, room setup and patient flow logistics were also addressed.

### Data-clinic evaluation measures

The following patient-level data were collected by chart review or clinical interview for each participant at the time of first visit: age, gender, use of durable medical equipment (e.g., cane, walker), falls in the last year, suspected PN etiology, CBOC location, previous neurology visit for PN within or outside the VA, previous PN labs (hemoglobin A1c or oral glucose tolerance test, vitamin B12, SPEP with IFE), previous nerve conduction study (NCS), previous PN imaging, previous PN medications, and previous referral to physical therapy for PN-related symptoms. Patient-reported travel time difference between local site of VA primary care and previous VA neurological site of care (or other VA specialist if no previous neurologic visit) was obtained by post-encounter interview.

To measure efficiency (goal 1) the neurologist recorded the duration of the encounter and exam at each visit.

To measure PN guideline-concordant care (goal 2) we recorded the medical decision making plan (e.g., tests, referrals, or treatments) from the health record and verified how often the neurologist completed three quality indicators: ordering all high-yield PN laboratory tests (any diabetes test, B12, SPEP) since the onset of PN symptoms [3]; reducing or eliminating opiate use for chronic non-cancer pain [23]; and ordering PT for patients with falls or near-falls in the last year [4, 24]. We selected this subset of guideline-concordant quality indicators based on institutional priorities and stakeholder feedback.

To measure patient satisfaction (goal 3) the study author conducted a short, verbal satisfaction survey (see Appendix) within 24 h after the appointment. In the survey, patients were asked about their satisfaction with the encounter using Likert scales (1–10 for overall score and 1–5 for component scores of vitals/check-in, exam, and history), their preference for follow-up visit location (a five-item ordinal rank from strong preference for usual in-person care at neurological site to strong preference for tele-PN care at local CBOC), and their feedback on what they liked most and least about the visit.

### Analysis

We provide summary statistics on key operational metrics of the tele-PN clinic: visit show-rate, travel time saved, encounter time, and exam time. We performed linear regressions of encounter and exam time on CBOC location, accounting for encounter type (initial vs. return). We performed Spearman correlations to look at the relationship between the pilot encounter number (listed as 1–48) and either the encounter time or exam time to look for trends in efficiency performance.

For care delivery, we first describe the baseline characteristics of the 25 patients. We then describe the percentage of guideline-concordant care in the tele-PN medical decision making plan.

For patient experience, we provide summary statistics for component satisfaction scores (i.e., check-in, exam, history), overall satisfaction, and clinic preference. We performed Spearman correlations to look at the relationship between the pilot encounter number (listed as 1–48) and satisfaction or clinic preference scores.

**Fig. 2:**
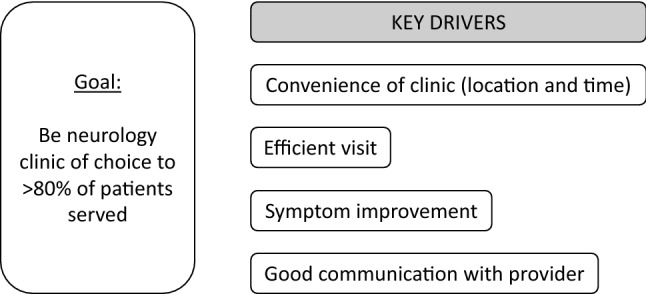
Key driver diagram. This diagram illustrates our proposed drivers of high-satisfaction scores, and ultimately, being the preferred clinical choice of patients who may either not receive specialty care, receive in-person specialty care at a more distant VA center, or receive specialty care outside of the VA system in their local area. This driver diagram includes structural factors (convenience of clinic), process factors (efficient visit, good communication), and outcome factors (symptom improvement) that we felt were important to measure to evaluate the clinic’s performance

We performed a thematic analysis with deductive coding on the free response survey feedback from patients and on performance improvement memos from the clinical team. The 4 a priori codes (convenience, efficiency, symptoms, and communication) were based on our key driver diagram for achieving high patient-satisfaction scores (Fig. 2), which was informed by Hebert’s telehealth evaluation framework [25]. For the qualitative analysis, a subset of authors (AMW, NIJ, AH, JAT) started with independent coding prior to meeting as a team to discuss, modify, and expand the codes. All authors reviewed and refined the final codes and themes.

Quantitative analysis was conducted using STATA v15. Statistical tests significant at the alpha = 0.05 level are listed in the results. Qualitative analysis was performed by hand.

## Results

### Tele-PN operational metrics

As of August 31, 2018, we had a sample of 48 scheduled encounters from the first 25 unique individuals. All 25 patients who were invited to participate in the pilot accepted the invitation to be seen in the tele-PN clinic. During the pilot phase, the show rate of the tele-PN clinic was 92% (44 out of 48 encounters). Compared to their usual site of neurologic or specialty care, the tele-PN clinic saved participants 231 min of round-trip travel per person with an interquartile range (IQR) of 150–280 min. The average encounter time (history, exam, and documentation) was 28.5 min (IQR of 22–32 min). The average exam time was 9.1 min (IQR of 8–10 min). Total encounter time was 8.8 min longer for initial than for return encounters (*P* value < 0.001). Total exam time was similar for initial and return exams. The pilot sites did not differ in their average encounter times or exam times. Total encounter time did not change over the study period, although exam duration declined over time (Spearman rho =  − 0.52; *P* value < 0.001), suggesting that more of the encounter was spent on non-exam activity.

### Tele-PN clinical care

#### Patient baseline characteristics

All patients were male with an average age of 70 years (SD 7 years). Six of the 25 patients had not previously been referred to VA neurology; 19 had seen a VA neurologist before, though 6 had been lost to follow-up. Eleven patients were using a mobility-assist device (e.g., cane, walker, or scooter). The three most common PN etiologies by medical documentation were diabetes/pre-diabetes (*n* = 11), medication-related (*n* = 5), and idiopathic (*n* = 5).

At baseline, complete high-yield lab testing (all three tests) had been performed for 20 patients since symptom onset. Eight patients were being chronically treated with short-acting opiates. Twelve patients had experienced falls in the last year, seven of whom had undergone physical therapy.

#### Quality indicators and updated plan of care

After their first tele-PN visit, the remaining five patients had the high-yield lab tests performed, bringing the percentage of patients receiving the entire high-yield set to 100%. Opiates were eliminated in 5 of 8 instances. Eighteen patients had consults placed for physical therapy for either balance/gait training (*n* = 13) or evaluation of a mobility-assist device (*n* = 5). All patients with recent falls were referred to physical therapy. The quality indicator performance is summarized in Table 2. Additional diagnostic and management changes are listed in the appendix.

**Table 2:** Guideline-concordant care indicators of the tele-PN pilot cohort

Quality indicator	Baseline performance	Post-pilot performance
All 3 high-yield PN tests completed^a^	80% (20/25)	100% (25/25)
Opiate freedom	68% (17/25)	88% (22/25)
Physical therapy in those with falls	58% (7/12)	100% (16/16)

The table highlights the performance on the three guideline-concordant care indicators of the tele-PN cohort at the time of their first visit and at the conclusion of the tele-PN pilot evaluation

P*N* polyneuropathy

^a^High-yield lab testing was evaluated after the first visit, rather than at the end of the pilot evaluation period

### Tele-PN patient experience

**Fig. 3:**
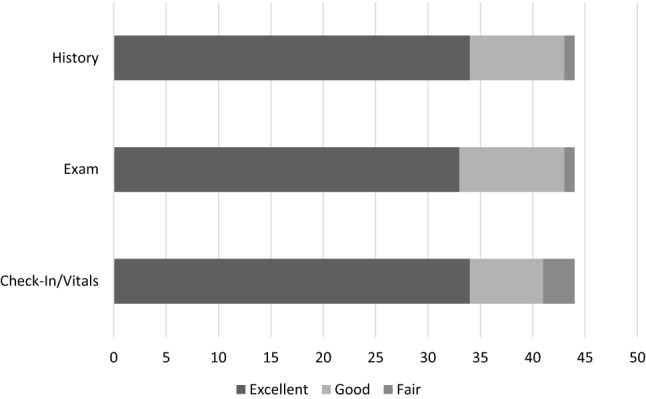
Patient satisfaction ratings by encounter component. For each encounter (*n* = 44), patients were asked to rate the check-in process, exam, and history on a 5-item scale from excellent to very poor. For the check-in process, there were 34 excellent, 7 good, and 3 fair ratings. For the exam, there were 33 excellent, 10 good, and 1 fair ratings. For the history, there were 34 excellent, 9 good, and 1 fair ratings. No respondents answered poor or very poor

Figure 3 summarizes the encounter-level, patient experience response for the check-in process, exam, and history. The average patient-satisfaction score (rated 1–10) of the clinic was 8.9 with an IQR of 8–10. When asked their preference of location for their future neurology visit, patients “strongly preferred” the tele-PN clinic 75% of the time (*n* = 33 encounters) and “slightly preferred” the tele-PN clinic 11% of the time (*n* = 5 encounters). No patient slightly or strongly preferred in-person care for follow-up. There was a significant Spearman correlation between the later chronological pilot encounter number and a more favorable ordinal follow-up preference rank (rho = 0.48; *P* value < 0.001). There was no significant Spearman correlation between the pilot encounter number and the overall satisfaction score or component satisfaction scores.

### Tele-PN qualitative feedback

Table 3 summarizes the themes and representative quotes that emerged from our content analysis of patients’ post-visit feedback and the clinical team’s monthly internal performance memos. As expected, the first theme focused on the convenient location of the clinic, which was frequently and favorably mentioned by patients. While the travel time reduction was substantial for all, we noticed that for some patients the convenience of tele-PN was simply a perk, while for others, it was a decisive factor in accessing neurologic care. This was especially true for patients with limited driving secondary to their medical conditions. The study team hypothesized that the limited time offering of the clinic would be a detraction, but patients were already accustomed to forgoing an entire day to be seen in a specialty clinic. Team memos do not reflect any challenges related to clinic timing.

**Table 3:**
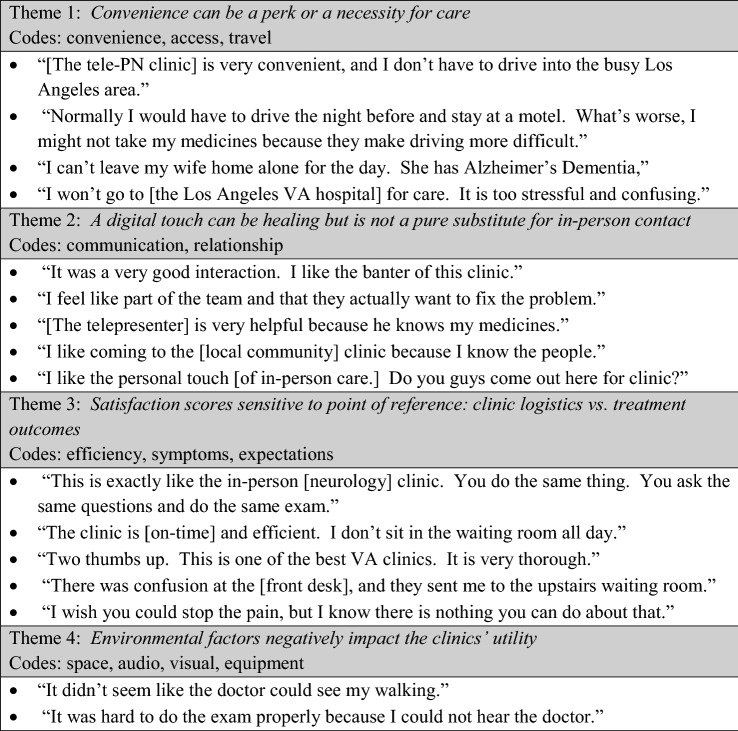
Qualitative themes and representative quotes

Another theme surrounded the interpersonal relationship between the patient and the care team. While some comments addressed the professionalism of the medical team, many comments revealed an affinity for the personality of the clinic. The memos reflect that Veterans perceived the tele-PN visits had a more casual and collaborative atmosphere. There were a similar number of comments about the neurologist and the telepresenter, highlighting an important contribution of the telepresenters beyond physical exam technicians. Despite the praise of the patient-care team relationship, many patients did ask if we were ever going to have an in-person clinic at the community site. We interpreted this to mean that the digital touch might not be an exact substitute for in-person contact with a specialist.

The third theme highlights the two distinct paradigms that patients used to rate the clinic. One set of patients focused on the mechanics of the tele-PN clinic and how it compared to other in-person clinics. Overall, this group was pleased with the efficiency of the clinic, with some variation in how the remote exam compared to in-person exams (a more structured and detailed exam versus exactly the same exam versus a simplified and abbreviated exam). The other set of patients focused on the clinical outcome of the visit. Patients who did not have substantive changes to the care plan, or who did not anticipate the changes to result in PN symptom improvement, provided more modest satisfaction scores.

Finally, the fourth theme focused on the environmental challenges to the tele-PN clinic, specifically the clinical space and technical aspects of the tele-PN encounter. In general, there were no unanticipated audiovisual issues. Patients with hearing loss did require more frequent repetition of questions and directions, which slightly reduced satisfaction scores. At two sites, the telemedicine room is small and requires additional maneuvering of the examination chair to complete the exam. This challenge was noted only once by a patient, whereas the team memos demonstrate considerable effort to conduct a more precise staging of the VANS exam within the room and a higher skill of camera steering to observe exam features. The smaller room was perceived to slow down the exam and potentially alienate patients, though not necessarily change the VANS score.

## Discussion

This study represents the first teleneurology pilot specifically designed for PN care. Over the course of 1 year, we were able to open and evaluate tele-PN clinics at three different CBOCs within the Greater Los Angeles VA region. We learned that these teleneurology clinics could be successfully incorporated into the larger healthcare ecosystem with a unique patient screening function to optimize the clinical utility of the clinic. At the initial visit, all patients were judged to have PN, and none were found to be inappropriate for the clinic. Further, we demonstrated at each spoke that the tele-PN care team (neurologist and telepresenter dyad) can see patients at least as efficiently as a normal in-person encounter. In fact, there is a suggestion from our timed encounters (nearly ¾ of which last less than 30 min) and patient feedback that our tele-PN visits are quicker than our in-person encounters, with a reduction in non-productive waiting time. This efficiency gain was not at the expense of desirable consultation time, which was praised as “thorough” and imperative for building a strong patient–doctor relationship. We also noted a lower no-show rate of 8% in the tele-PN clinics compared to 10–15% for our in-person clinic (personal communication with clinic manager), despite using the same appointment reminders as our in-person clinic. Whether this was related to a small sample size, the novelty and convenience of the clinic, or another factor is not known.

Similar to previous outpatient teleneurology studies [26–28], our pilot consistently received excellent satisfaction scores. Patients preferred to follow-up in the tele-PN clinic over our closest in-person neurology clinic 86% of the time, with the remaining 14% having no preference. As expected, convenience was a big driver of its favorability, saving an average travel time of 231 min per patient; however, we also explicitly asked about the encounter components, and the check-in process, exam, and history were each rated as “very good” or “excellent” about 95% of the time. In particular, patients appreciated “smooth” transitions and the personal touch of the tele-PN clinic. The theme of strong interpersonal engagement as a marker of quality in teleneurology has been noted elsewhere [29]. Of import, the care team memos also reflect a joy for this team-based care and the diversity it brings to their other clinical tasks. Concerns with the telemedicine technology were largely absent from patient feedback and provider memos, though it appears patients who are hard of hearing may be susceptible to a subpar experience. We speculate that the remote encounter may slightly distort the audio and make it more difficult to lip-read. Interestingly, while smaller telemedicine rooms were felt to hinder the VANS exam by the provider team, longer exam times or lower satisfaction scores were not found in the pilot.

Importantly, our tele-PN clinic was able to improve the care of patients with moderate-to-severe PN (mild PN was not well represented in the pilot). This cohort had many risk factors for deterioration including advanced age, polypharmacy, falls, and the need for mobility-assist devices. In only three cases did the neurologist not order additional diagnostic tests, make a referral, or adjust medications. In all other cases, there was at least one shared-decision management change. In terms of guideline-concordant care for the cohort, high-yield labs were ordered for five patients (leading to two new diagnoses and a 100% compliance rate for lab testing), timely referrals to physical therapy were made for all 16 patients with falls, and opiates were successfully reduced or replaced with preferred neuropathic pain medicines in five of eight patients. Beyond these pre-specified indicators, there were numerous other instances of improved pain control with more aggressive treatment. While most patients did see improvement in their symptoms, the continued existence of this chronic condition did leave some requesting even more radical therapies (e.g., amputation, stem cells).

Given the disparity between the short supply of neurologists, especially in rural areas, and the high prevalence of PN, teleneurology clinics tailored for PN could bridge this gap in access to care. The hub-and-spoke model of our telemedicine pilot study is well suited to enabling a specialist in an urban setting to evaluate and manage patients with common disorders who live in remote or medically underserved regions. Future investigations could explore the feasibility of large-scale tele-PN clinics and their ability to improve neurological care in the setting of a limited workforce of neurologists.

## Limitations

As a telemedicine pilot study expanding to a new condition, this work has some expected limitations. We present rich clinical data on a small sample of 25 patients without a clinical control. The patient selection was not random, but driven by geographic and clinical factors. We believe these patients to be practical and informative “real-world” test cases for tele-PN. The fact that a large majority of our sample had seen neurology in-person before is typical of our health system, and likely diminishes the impact the tele-PN clinic would have on guideline-concordant care in neurology-naïve or de novo cases. The scope of this work is also limited—we do not address clinical outcomes (including patient-reported outcomes), healthcare utilization, or costs. We hope to evaluate these factors in future quasi-experimental and experimental approaches.

The tele-PN clinic has important contextual features that impact its generalizability. The VA-integrated health system has unique financial and health care delivery structures that support telemedicine. Accessible medical records and existing telemedicine equipment and personnel were essential to the successful screening and management of patients with PN for this pilot. Healthcare systems without these features may not be able to replicate tele-PN clinics in the same fashion as we present here. Also, as currently constructed, the tele-PN clinics do not impact individuals in our health system who may have signs or symptoms of PN, but do not carry the diagnosis in the electronic health record. Additional studies of how telehealth initiatives, including telemedicine, fit into a larger evidence-based practice environment is still warranted.

Finally, we (AMW, NIJ, AH) acknowledge our own potential biases to act as both clinicians and researchers in this evaluation. Where possible, we have created independent review of tasks requiring clinical judgement, and we openly discuss our findings with the entire research team in pursuit of a fair and informative evaluation of this pilot study.

## Conclusion

In conclusion, we show that teleneurology for polyneuropathy is feasible in the setting of thoughtful patient screening and remote neurologic examination. Based on our mixed-methods pilot evaluation, tele-PN clinics demonstrate promise as a promoter of evidence-based practice and a patient-preferred mode of care. Given this feasibility and acceptability, future studies should focus on demonstrating their efficacy in improving the patient experience, improving health outcomes, and reducing costs compared to the standard of care. We hope this work will stimulate more consideration of how teleneurology can be utilized to improve the care pathway for patients with neurologic disorders.

## Appendix 2

See Appendix Table 4.

**Table 4:** Additional clinical characteristics of the pilot

Characteristic	Baseline performance	Pilot performance
Diagnostic tests	–	–
All 3 high-yield PN tests^a^	80%	100%
Nerve conduction study	76%	76%
Lumbosacral MRI	32%	32%
PN medication, current	–	–
Gabapentinoids	64%	76%
SNRIs	16%	20%
Opiates	32%	12%
TCAs	4%	8%
Carbamazepine	0%	4%
Topicals	16%	48%
Falls in last year	48%	64%
With physical therapy	28%	64%
Without physical therapy	20%	0%
Physical therapy in last year	44%	72%
PN Etiology (not mutually exclusive)	–	–
Diabetes/pre-diabetes	40%	44%
Medication-related	20%	20%
Idiopathic	20%	16%
Alcohol-related	12%	12%
Environmental	12%	12%
Gammopathy	4%	8%
Inflammatory	8%	8%
Viral	8%	8%
Hereditary	4%	4%
Vitamin deficiency	0%	0
Durable medical equipment	–	–
Cane	28%	32%
Walker	12%	20%
Scooter	4%	8%

The table highlights the main clinical care features of the tele-PN cohort at the time of their first visit and at the conclusion of the tele-PN pilot evaluation. The table notes changes in utilization of diagnostic tests, PN medication, and physical therapy. The table also notes changes in falls, durable medical equipment needs, and etiologic diagnosis for PN

*PN* polyneuropathy,* SNRIs* serotonin norepinephrine reuptake inhibitors,* TCAs* tricyclic antidepressants

^a^High-yield lab testing was evaluated after the first visit, rather than at the end of the pilot evaluation period
